# Loss of MeCP2 in adult 5-HT neurons induces 5-HT1A autoreceptors, with opposite sex-dependent anxiety and depression phenotypes

**DOI:** 10.1038/s41598-018-24167-8

**Published:** 2018-04-10

**Authors:** Tristan J. Philippe, Faranak Vahid-Ansari, Zoe R. Donaldson, Brice Le François, Amin Zahrai, Valérie Turcotte-Cardin, Mireille Daigle, Jonathan James, René Hen, Zul Merali, Paul R. Albert

**Affiliations:** 1 Ottawa Hospital Research Institute (Neuroscience), University of Ottawa Brain and Mind Research Institute, Ottawa, ON Canada; 2 Department of Cellular and Molecular Medicine, University of Ottawa Brain and Mind Research Institute, Ottawa, ON Canada; 3 Department of Molecular, Cellular, and Developmental Biology and Department of Psychology & Neuroscience, University of Colorado Boulder, Boulder, CO USA; 4 New York State Psychiatric Institute, Columbia University Medical Center and Research Foundation for Mental Hygiene, New York, NY USA; 5 Department of Psychiatry, Columbia University, New York, NY USA; 6 The Royal’s Institute of Mental Health, affiliated with the University of Ottawa, Ottawa, ON Canada

## Abstract

The 5-HT1A autoreceptor mediates feedback inhibition of serotonin (5-HT) neurons, and is implicated in major depression. The human 5-HT1A gene (HTR1A) rs6295 risk allele prevents Deaf1 binding to HTR1A, resulting in increased 5-HT1A autoreceptor transcription. Since chronic stress alters HTR1A methylation and expression, we addressed whether recruitment of methyl-binding protein MeCP2 may alter Deaf1 regulation at the HTR1A locus. We show that MeCP2 enhances Deaf1 binding to its HTR1A site and co-immunoprecipitates with Deaf1 in cells and brain tissue. Chromatin immunoprecipitation assays showed Deaf1-dependent recruitment of MeCP2 to the mouse HTR1A promoter, and MeCP2 modulated human and mouse HTR1A gene transcription in a Deaf1-dependent fashion, enhancing Deaf1-induced repression at the Deaf1 site. To address the role of MeCP2 in HTR1A regulation *in vivo*, mice with conditional knockout of MeCP2 in adult 5-HT neurons (MeCP2 cKO) were generated. These mice exhibited increased 5-HT1A autoreceptor levels and function, consistent with MeCP2 enhancement of Deaf1 repression in 5-HT neurons. Interestingly, female MeCP2-cKO mice displayed reduced anxiety, while males showed increased anxiety and reduced depression-like behaviors. These data uncover a novel role for MeCP2 in 5-HT neurons to repress HTR1A expression and drive adult anxiety- and depression-like behaviors in a sex-specific manner.

## Introduction

Methyl binding proteins, such as MeCP2, are highly expressed in the brain^1^ and recognize epigenetic DNA methylation marks to repress gene transcription^2^. However, MeCP2 can also enhance transcription, in part through interaction with other transcription factors^3^. MeCP2 mutations have been associated with Rett syndrome^4^, while MeCP2 gene duplication associates with autism and an increased prevalence of anxiety, depression, and compulsions in patients^5^. Patients with Rett syndrome have reduced levels of monoamine metabolites^6^, similar to MeCP2 knockout mice that also display increased anxiety^7^. These results suggest that MeCP2 also plays a role in mood disorders.

Depression is a complex mental health disorder that affects 16–20% of the population^8,9^. Depressed patients and suicide attempters were shown to have reduced serotonergic activity^10–12^, are more susceptible to tryptophan depletion^13,14^, and have lower 5-HT metabolites in their cerebrospinal fluid^15,16^. The 5-HT1A autoreceptor of the raphe nuclei inhibits pre-synaptic neuronal firing and reduces 5-HT release to negatively regulate 5-HT neurotransmission in the brain^17^. Elevated 5-HT1A autoreceptor levels have been associated with major depression and suicide, as observed in human post-mortem studies^18,19^ and PET imaging studies^20,21^. Conversely, decreases in post-synaptic 5-HT1A heteroreceptors in prefrontal cortical areas have been associated with anxiety and depression^22–25^. Conditional 5-HT1A knockout and 5-HT1A transgenic mouse models have demonstrated that changes in the level of 5-HT1A receptors pre- and post-synaptically and during development can lead to anxious, depressed, or anti-depressed phenotypes^26,27^. However, the transcriptional mechanisms regulating brain-region specific 5-HT1A receptor levels *in vivo* are only beginning to be clarified^28,29^.

In humans, altered regulation of the 5-HT1A gene (HTR1A) in depression was first suggested when we identified the association of the C/G(-1019) rs6295 HTR1A polymorphism, located in the 5-HT1A promoter region^30^. The association of the G(-1019) allele with depression has been replicated^31,32^, and it is also associated with other disorders including anxiety, suicide, and substance abuse^33–35^. The rs6295 polymorphism is located within an imperfect 26-bp palindrome, which is recognized and bound by Deaf1 at the C-allele, but not the G-allele^30,36^. Impaired recruitment of Deaf1 to the HTR1A gene in 5-HT neurons is expected to increase 5-HT1A autoreceptor levels^30^, consistent with studies showing that the G-allele is associated with increased 5-HT1A autoreceptor binding in the raphe of depressed patients^21,37^. In Deaf1 knockout mice, loss of Deaf1 led to a functional increase in 5-HT1A autoreceptors and an anxiety phenotype in males and females^28,38^. Conversely, the loss of Deaf1 also led to a decrease in cortical 5-HT1A receptors, by preventing Deaf1 enhancer function that is seen in non-serotonergic neuronal cells^36^. These data suggest that Deaf1 bidirectionally regulates the HTR1A gene in different brain regions but is unable to do so at the G(-1019) variant.

In addition to genotype risk, evidence indicates that the 5-HT1A-G/G genotype is more strongly associated with psychopathology in individuals subject to environmental stressors^34,39–41^. While the mechanisms of this gene-environment interaction remain elusive, early life or adult stress has been associated with changes in site-specific DNA methylation that alter transcription factor binding, resulting in persistent changes in gene expression^42–46^. The Deaf1 site in the human 5-HT1A gene contains two potential CpG DNA methylation sites, one of which is more frequently methylated in schizophrenia and is associated with negative symptoms^47^. The effect of DNA methylation on Deaf1 binding to this site is not known but would be predicted to reduce on Deaf1 binding^48^. Thus, we studied the potential role of MeCP2 in regulating HTR1A gene expression, its interaction with Deaf1, and the functional effects of conditionally knocking out MeCP2 in 5-HT-producing neurons on 5-HT1A autoreceptor expression. Our findings indicate that while Deaf1 and MeCP2 interact to regulate 5-HT1A receptor gene expression in 5-HT neurons, MeCP2 also has sex-dependent actions that impact behavior.

## Results

### Deaf1-MeCP2 interaction enhances recruitment to the HTR1A promoter

**Figure 1:**
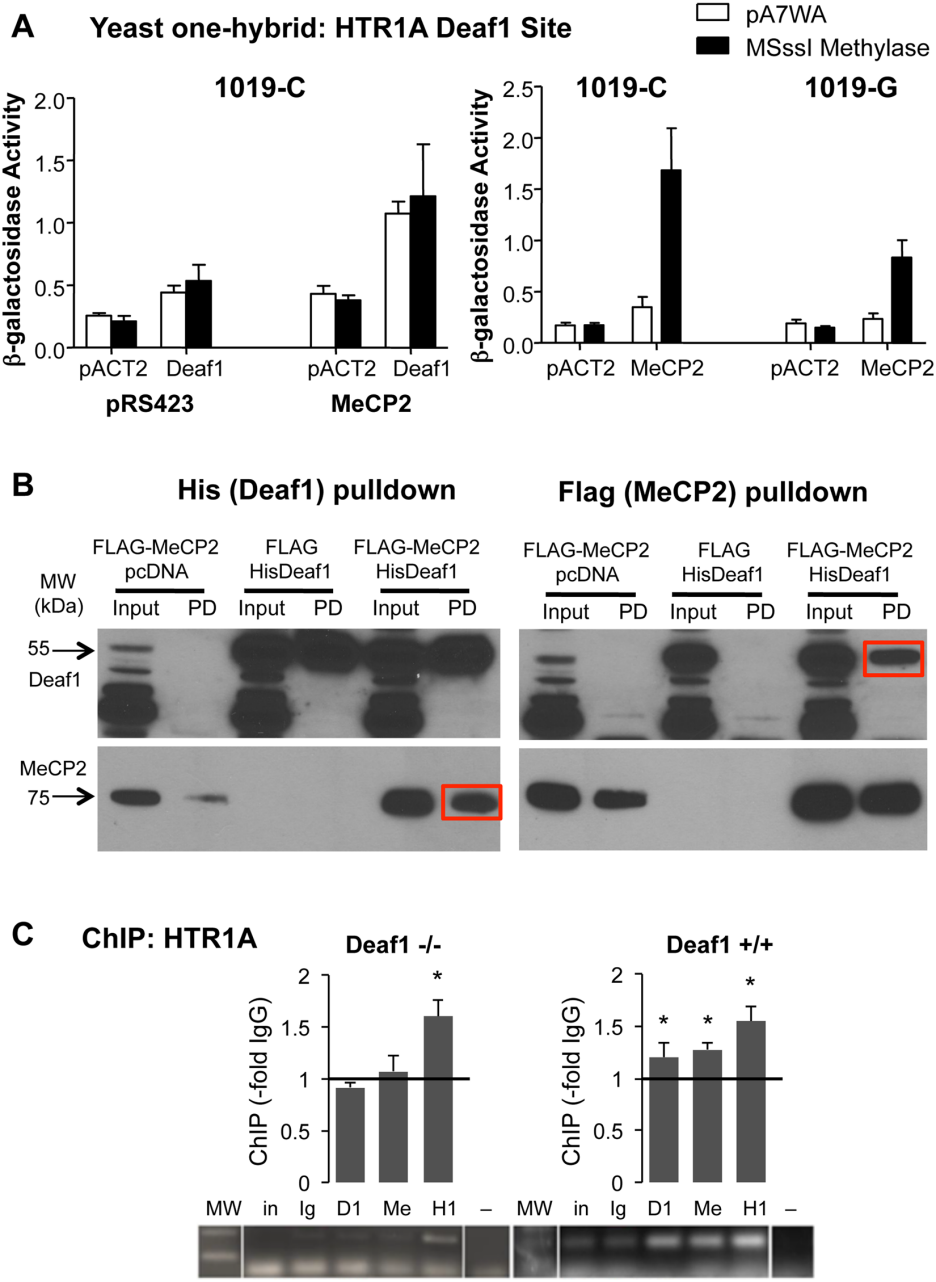
Deaf1-MeCP2 interaction enhances their recruitment to the HTR1A promoter Deaf1 site. (**A**) MeCP2 increases Deaf1 transactivation of the human HTR1A C(-1019) site independent of promoter methylation. At left, yeast were transduced with a combination of Deaf1-GAL4AD or vector (pACT2), MSssI or vector (pA7WA), and MeCP2 or vector (pRS423) and the β-Gal gene fused to three copies of the human 5-HT1A 26 bp Deaf1 site with the C (1019-C) in the p8op vector. The level of β-galactosidase activity is shown as a measure of transactivation. At right, yeast were transduced with a combination of MeCP2-GAL4AD or vector (pACT2), MSssI or vector, and either the C or G allele of the HTR1A Deaf1 element (1019-C or 1019-G). Data shown as mean ± S.E., n = 3. (**B**) His-Deaf1 and Flag-MeCP2 co-precipitate in co-transfected human SKN-SH neuroblastoma cells. His-Deaf1, FLAG-MeCP2 or FLAG-vector were co-transfected in HEK-293 cells and cell extracts (Input) analyzed by pull-down (PD) followed by Western blot for Deaf1 or FLAG (MeCP2). Left, FLAG-MeCP2 but not FLAG was enriched upon of His-Deaf1; right, His-Deaf1 was enriched upon pull-down of FLAG (red boxes). (**C**) Deaf1 recruits MeCP2 to the Deaf1 sites on the mouse HTR1A promoter. Deaf1 −/− and +/+ MEF extracts were analyzed by ChIP using pull-down with pre-immune rabbit IgG (Ig), n = 8; Deaf1 antibody (D1), n = 8; MeCP2 antibody (Me), n = 4; or histone H1 antibody (H1), n = 4. PCRs of precipitated DNA were run on 2% agarose gel. Above, HTR1A promoter ChIP. The mean total luminosity within a standard band template was quantified and normalized to the appropriate pre-immune IgG and plotted as –fold IgG ± SE, *p < 0.05. Below: Representative PCR result showing the 200-bp and 100-bp bands of the molecular size ladder (MW), input (in) and a negative (buffer) PCR control (−).

Because methylation of the Deaf1 site of the 5-HT1A gene is associated with negative symptoms of schizophrenia, we addressed whether its methylation affects Deaf1 and/or MeCP2 recruitment, since MeCP2 binds to methylated DNA. The 26-bp human 5-HT1A Deaf1 element was inserted into a yeast one-hybrid vector and β-galactosidase activity was measured as a read-out for recruitment of Deaf1 or MeCP2 (Fig. 1A). Since yeast lack endogenous DNA methylase, the DNA methylase transferase MSssI was transduced for comparison of methylated and non-methylated states. Transduction of Deaf1 increased β-galactosidase activity at the C-allele, independent of DNA methylation, an effect that was enhanced in the presence of MeCP2 (Fig. 1A, left). When expressed alone, MeCP2 was not recruited to the Deaf1 site unless it was methylated and showed significantly more interaction at the C-allele, which contains two CpG sites compared to one site in the G-allele (Fig. 1A, right). This result suggests that MeCP2 can enhance Deaf1 recruitment to its site independent of DNA methylation, but in the absence of Deaf1, MeCP2 is recruited to the Deaf1 site only upon its methylation.

To determine whether MeCP2 and Deaf1 proteins interact, pull-down assays were done in human 5-HT1A-expressing SKN-SH neuroblastoma cells co-transfected with Flag-MeCP2 and His-Deaf1 (Fig. 1B). Pull down of His-tagged Deaf1 co-precipitated Flag-MeCP2, with weak background signal in the absence of His-Deaf1 (Fig. 1B, left). Conversely, pull down of Flag-tagged MeCP2 resulted in His-Deaf1 precipitation (Fig. 1B, right). To confirm the Deaf1-MeCP2 interaction, IgG, Deaf1, or MeCP2 antibodies were used to immunoprecipitate endogenous proteins from extracts of SKN-SH cells, hippocampal tissue or MEFs from Deaf1 knockout or wild-type mice. MeCP2 was detected in anti-MeCP2 precipitates in all samples, and was enriched when pulling down for Deaf1 in SKN-SH, Deaf1 +/+ MEF and WT hippocampal extracts, but not in Deaf1 −/− MEFs and Deaf1 KO hippocampal tissue (Supplementary Fig. S1). It was not possible to detect Deaf1 due to cross-reactivity with IgG heavy chain, and the lack of a suitable alternate Deaf1 antibody. Taken together, these results indicate that MeCP2 and Deaf1 proteins interact.

To examine whether Deaf1 affects MeCP2 binding to the HTR1A promoter *in vivo*, we performed chromatin immunoprecipitation (ChIP) assays in the Deaf1 +/+ and −/− MEF cell lines followed by PCR for the two Deaf1 sites of the mouse HTR1A promoter^38^ (Fig. 1C). Immunoprecipitation of Deaf1 and MeCP2 significantly enriched the HTR1A promoter region in the Deaf1 +/+ MEFs (t(2) = 6.32, p = 0.0071 and t(2) = 6.132, p = 0.0133; post hoc: p = 0.022; p = 0.007, respectively), but not the Deaf1 −/− MEFs (Fig. 1C; post hoc: p = 0.787; p = 0.568, respectively). Immunoprecipitation of Histone H1, a ubiquitous component of chromatin, was used as a positive control and significantly enriched the site in Deaf1 +/+ and −/− MEFs (Fig. 1C; t(2) = 7.202, p = 0.0078; post hoc p = 0.0212 and p = 0.0074, respectively). The GAPDH gene, a negative control, was only enriched when pulling down with histone H1 (data not shown). To substantiate this result, similar experiments were performed in 5-HT1A-enriched brain regions including raphe, hippocampal, and prefrontal cortex (PFC) tissues from Deaf1 WT and KO mice (Supplementary Fig. S2). Pull down for Deaf1 and MeCP2 significantly enriched the site only in the Deaf1 WT mice. Overall, these data indicate that Deaf1 binds to the Deaf1 site *in vivo* and is required for MeCP2 binding to this site in mice.

### Deaf1 and MeCP2 modulate human and mouse HTR1A promoter activity

**Figure 2:**
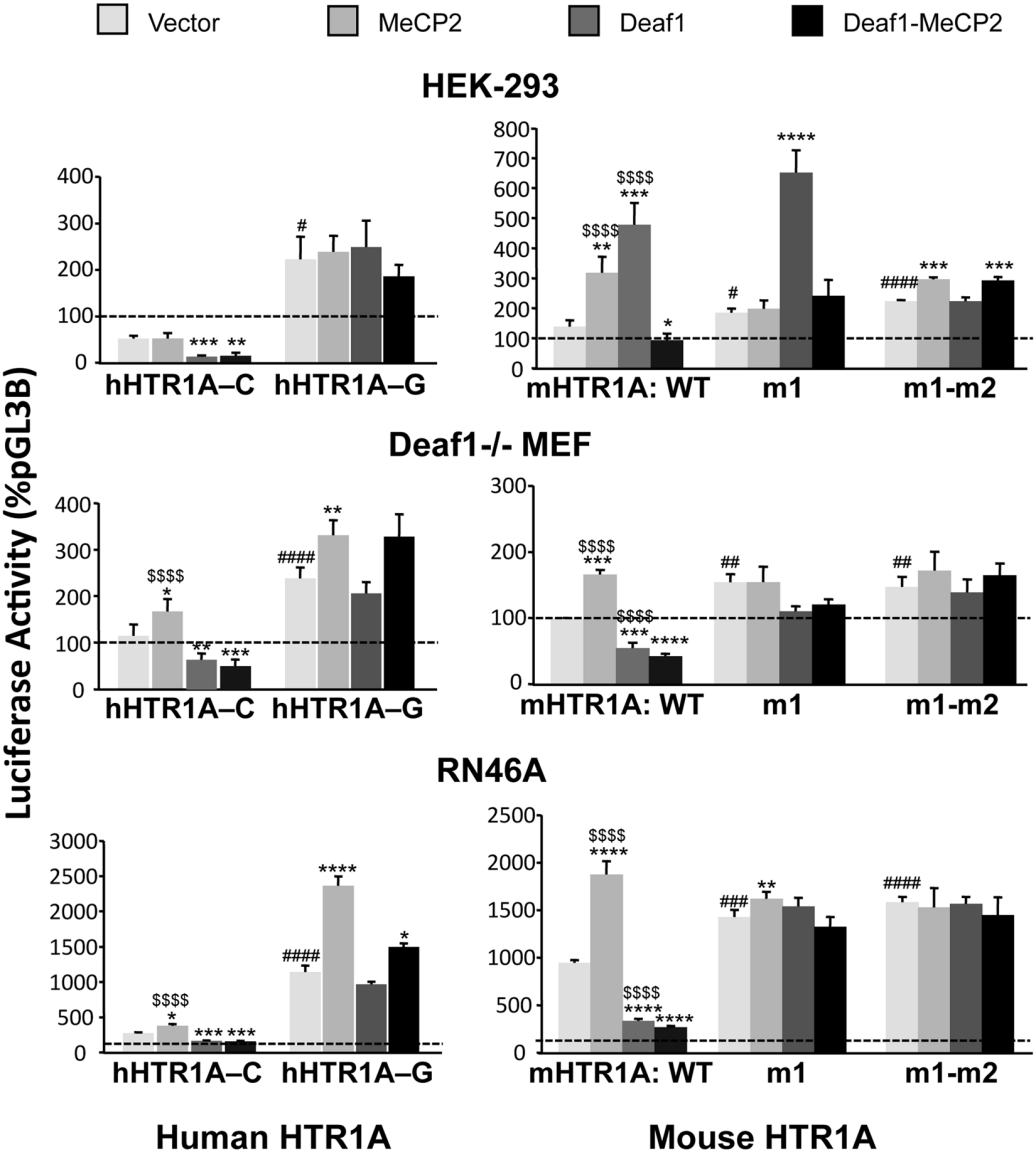
Activity of Deaf1 and MeCP2 at human or mouse HTR1A promoter Deaf1 site mutants. Cell lines were co-transfected with Deaf1 and/or MeCP2 constructs or appropriate empty vector controls, β-Galactosidase (β-Gal) plasmid, and either (at left) the human 1128 C or 1128 G HTR1A promoters (hHTR1A-C or -G, respectively) or (at right) mouse HTR1A promoter constructs wild-type (WT) or mutants of Deaf1 site 1 (m1) or both Deaf1 sites 1 and 2 (m1-m2). Luciferase values were normalized to β-Gal, then normalized to the empty pGL3B vector and expressed as –fold pGL3B luciferase activity. β-Gal activities were not significantly different between samples for co-transfection of pGL3B with MeCP2 and/or Deaf1 or empty vector (data not shown). Results from HEK-293 (n = 12), Deaf1−/− MEF (n = 9), and RN46A cells (n = 9) are plotted as Mean ± SE. *Deaf1, MeCP2, or both vs. vector; ^#^vector HTR1A-G vs. C or mutant vs. WT; ^$^Deaf1 or MeCP2 alone vs. Deaf1-MeCP2. *^,#,$^p < 0.05; **^,##,$$^p < 0.01, ***^,###,$$$^p < 0.001; ****^,####,$$$$^p < 0.0001 (see Supplementary Tables 1, 2 for statistics).

In order to address whether the Deaf1-MeCP2 interaction has functional consequences, we examined their impact on the regulation of human and mouse HTR1A transcriptional activity in HEK-293, Deaf1−/− MEFs, and raphe RN46A cell lines using luciferase reporter assays (Fig. 2). Rat raphe RN46A cells model Deaf1 repression of 5-HT1A autoreceptor transcription in 5-HT neurons^30,36^, the HEK-293 cells are a human non-neuronal cell type that lacks 5-HT1A receptors^36,49^ and the mouse Deaf1−/− fibroblasts provide a Deaf1-deficient background^38^.

For the human HTR1A promoter, we compared the action of Deaf1, MeCP2, or both at the 1128-C or 1128-G luciferase expression vectors (Fig. 2 left panels, Table 1). MeCP2 significantly enhanced the activity of the 1128-C and 1128-G constructs in Deaf1 −/− MEF and RN46A cells, but had no detectable effect in HEK293 cells. These results suggest that MeCP2 has enhancer activity at the human HTR1A promoter that does not require Deaf1 or an intact Deaf1 site. While Deaf1 significantly repressed 1128-C activity, this effect was lost in the 1128-G variant consistent with the inability Deaf1 to bind to the G-allele^30^. Co-transfection of Deaf1 and MeCP2 significantly repressed 1128-C in all cell types, but significantly enhanced 1128-G activity in the Deaf−/− MEF and RN46A but not HEK-293 cells. Therefore, it appears that Deaf1 can only overcome the enhancer activity of MeCP2 in the presence of its functional site. Furthermore, the 1128-G variant was significantly de-repressed compared to the 1128 C in all cell types (HEK-293: t(8) = 3.423, p = 0.009; Deaf1 −/− MEF: t(8) = 4.247, p = 0.0003; RN46A: t(8) = 10.27, p < 0.0001). These data confirm that Deaf1 cannot repress the 1128-G allelic variant.

**Table 1:** Summary of the effects of Deaf1 and MeCP2 on activity of the human HTR1A promoter.

	1128-C			1128-G		
	M	D	MD	M	D	MD
HEK 293	1	**−3.8**	**−3.3**	1	1	1
MEF Deaf1−/−	**+1.5**	**−1.8**	**−2.3**	**+1.5**	1	**+1.5** ^**#**^
RN46A	**+1.4**	**−1.6**	**−1.7**	**+2.1**	1	**+1.3**

Shown is the -fold effect of Deaf1 (D), MeCP2 (M) or both (MD) on 1128-C and 1128-G HTR1A promoter activity in luciferase reporter assays, normalized to the expression vector with empty Deaf1 and MeCP2 vector controls (1x). Significant (p < 0.05) increases (+) and decreases (−) are in bold. ^#^p < 0.10.

The function of MeCP2 and Deaf1 at the mouse HTR1A promoter was assessed by comparing wild-type and Deaf1 site mutant HTR1A promoters (Fig. 2 right panels, Table 2). Since the mouse HTR1A promoter contains two potential Deaf1 binding sites^38^, we mutated site 1 (m1), which is critical for Deaf1 repression^38^, or both Deaf1 sites 1 and 2 (m1-m2). In HEK293 cells, Deaf1 enhanced the activity of WT and m1 but not the m1-m2 mutant, suggesting a role for site 2 in Deaf1 enhancer activity (Table 2). In RN46A and Deaf1 −/− MEF cells, Deaf1 repressed WT activity, but not m1 and m1-m2 activity, indicating a role for HTR1A Site 1 in Deaf1 repressor activity, as observed previously using deletion mutants^38^. In all cell lines, MeCP2 exhibited enhancer activity that was blocked in the m1 or m1-m2 mutant construct, suggesting that the enhancer action of MeCP2 is dependent on Deaf1 sites. The combination of Deaf1 and MeCP2 significantly repressed the WT promoter, suggesting a dominant effect of Deaf1 repressor activity. Interestingly, in HEK293 cells, the enhancer effects of both MeCP2 and Deaf1 were converted to a repressor effect with the combination. These repressor effects of the MeCP2-Deaf1 combination were not seen in the m1 or m1-m2 mouse HTR1A promoter variants, consistent with the importance of site 1 in Deaf1 repressor function.

**Table 2:** Summary of the effects of Deaf1 and MeCP2 on activity of the mouse HTR1A promoter

	WT			m1			m1&m2		
	M	D	MD	M	D	MD	M	D	MD
HEK 293	**+2.3**	**+3.4**	**−1.5**	1	**+3.5**	1	+1.2	1	+1.2
MEF Deaf1−/−	**+1.7**	−1.8	**−2.4**	1	1	1	1	1	1
RN46A	**+2.0**	**−2.8**	**−3.5**	+1.1	1	1	1	1	1

Shown is the -fold effect of Deaf1 (D), MeCP2 (M) or both (MD) on wild-type (WT) and mutants at site 1 (m1) and sites 1 and 2 (m1&m2) mouse HTR1A promoter activity in luciferase reporter assays, normalized to the expression vector with empty Deaf1 and MeCP2 vector controls (1x). Significant (p < 0.05) increases (+) and decreases (−) are in bold.

### MeCP2 cKO mice have higher 5-HT1A autoreceptor levels and function

**Figure 3:**
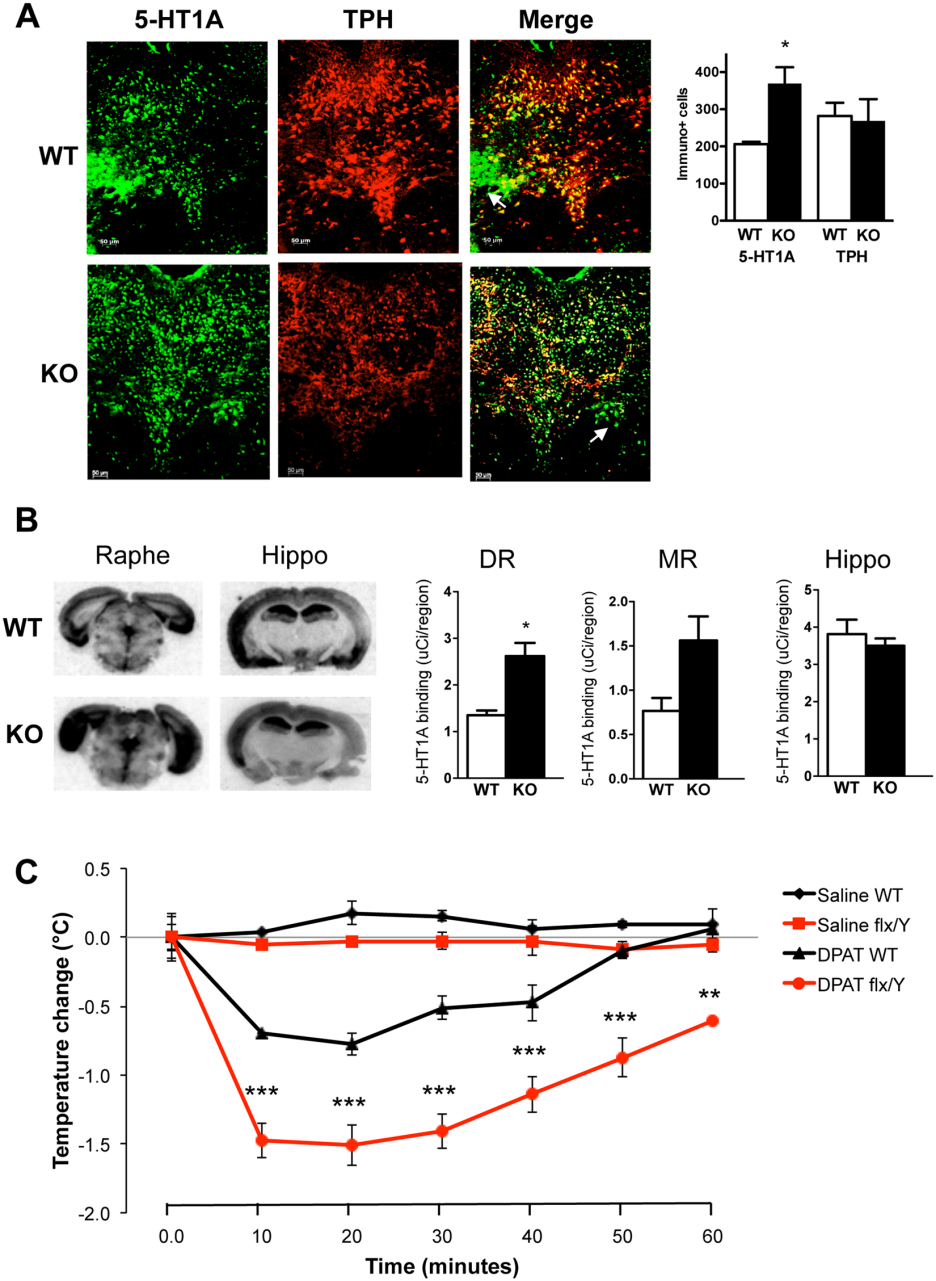
Increased 5-HT1A autoreceptor levels and function in MeCP2 cKO mice. Male TPH2^creERT2^-MeCP2flx/Y conditional knockout (KO) and control littermates (WT) mice were treated with tamoxifen at 8 weeks of age and perfused three weeks later. **(A**) Dorsal raphe slices were stained with TPH (sheep anti-TPH, 1:100) and 5-HT1A receptors (rabbit anti-5-HT1A, 1:50). Representative sections are shown at left; arrows show a small group of 5-HT1A + /TPH-negative cells; scale bar indicates 50 μm. Right, total 5-HT1A- and TPH-positive cells were counted in dorsal raphe sections and quantified (n = 3 animals) as mean ± SEM (for 5-HT1A: WT, 206 ± 6.5 vs. KO, 369 ± 43.8, t test, t = 3.691732 df = 4, *p = 0.0210; for TPH, WT, 281.7 ± 35.8 vs cko, 268.7 ± 58.4, t test, t = 0.1898 df = 4, p = 0.8587). (**B**) 5-HT1A receptor autoradiography. ^125^I-MPPI autoradiography was performed using raphe (containing dorsal (DR) and median (MR)) and hippocampal (Hippo) slices (representative sections shown at left). At right, signal/region template was quantified as mean ± SEM (n = 3) (for DR: WT, 1.35 ± 0.10 vs cko, 2.62 ± 0.28, t test, t = 4.244 df = 4, *p = 0.0132; for MR: WT, 0.768 ± 0.145 vs cko, 1.56 ± 0.273, t test, t = 2.563 df = 4, p = 0.0625; for hippocampus, WT, 3.81 ± 0.386 vs cko, 3.50 ± 0.195, t test, t = 0.7246 df = 4, p = 0.5088). **(C)** DPAT-induced hypothermia. Mice were injected with 0.9% saline (vehicle) or 0.5 mg/kg 8-OH-DPAT at 0 min (temperature change = 0) and rectal temperatures measured at 10-min intervals thereafter, averaged and subtracted from mean pre-injection temperature, over a 60-min time period (n = 5 WT; n = 3 Flx/Y KO). Data are shown as mean ± SE; **p < 0.01; ***p < 0.001 by two-way ANOVA with Bonferonni post-test, comparing DPAT-treated Flx/Y to WT.

Previously we showed that Deaf1 -/- mice have increased 5-HT1A autoreceptors, reduced raphe 5-HT levels and are more anxious than their wild-type littermates^28,38^. To address the impact of MeCP2 on 5-HT1A receptor regulation and the 5-HT system *in vivo*, we generated mice with a conditional knockout of MeCP2 in 5-HT neurons (MeCP2^TPH2 creERT2-flx/Y^), and induced this knockout in adulthood to avoid developmental complications. Immunofluorescence co-labeling of the 5-HT1A receptors and TPH2 (5-HT neuron marker) in the raphe of MeCP2^TPH2 creERT2-MeCP2/Y^ (WT) and MeCP2^TPH2 creERT2-flx/Y^ (KO) male mice revealed an almost 2-fold increase in 5-HT1A-labelled cells, with >90% of these TPH/5-HT1A co-labeled (Fig. 3A). A small proportion of 5-HT1A-stained cells were TPH-negative, which may reflect GABAergic interneurons, known to express 5-HT1A receptors (white arrow). In order to quantify the level of functional 5-HT1A binding sites, brain sections were analyzed by autoradiography analysis using ^125^I-MPPI, a selective 5-HT1A antagonist (Fig. 3B). In the dorsal raphe there was a significant two-fold increase in 5-HT1A binding levels, and a similar two-fold trend in the median raphe in the knockout brains. By contrast, the hippocampus, in which the MeCP2 gene was not targeted, showed no change in the level of 5-HT1A receptor binding. These data indicate that the loss of MeCP2 induces an increase in 5-HT1A autoreceptors, but no change in post-synaptic 5-HT1A receptors. DPAT-induced hypothermia was assessed, since in mice it provides a read-out of the functional activity of 5-HT1A autoreceptors *in vivo*. Acute injection of 8OH-DPAT, but not vehicle (saline), caused a significant reduction in temperature in both control (WT) and knockout (flx/Y) male mice, but the drop in temperature was greater in the knockout compared to controls at all times measured (Fig. 3C; F(11,36) = 21.234, p < 0.0001). Taken together, these results indicate that loss of MeCP2 in 5-HT neurons leads to increased 5-HT1A autoreceptor levels and function, consistent with a role for MeCP2 to promote repression of the HTR1A gene in serotonergic neurons *in vivo*.

### Altered anxiety and depression-like behavior in MeCP2 cKO mice

**Figure 4:**
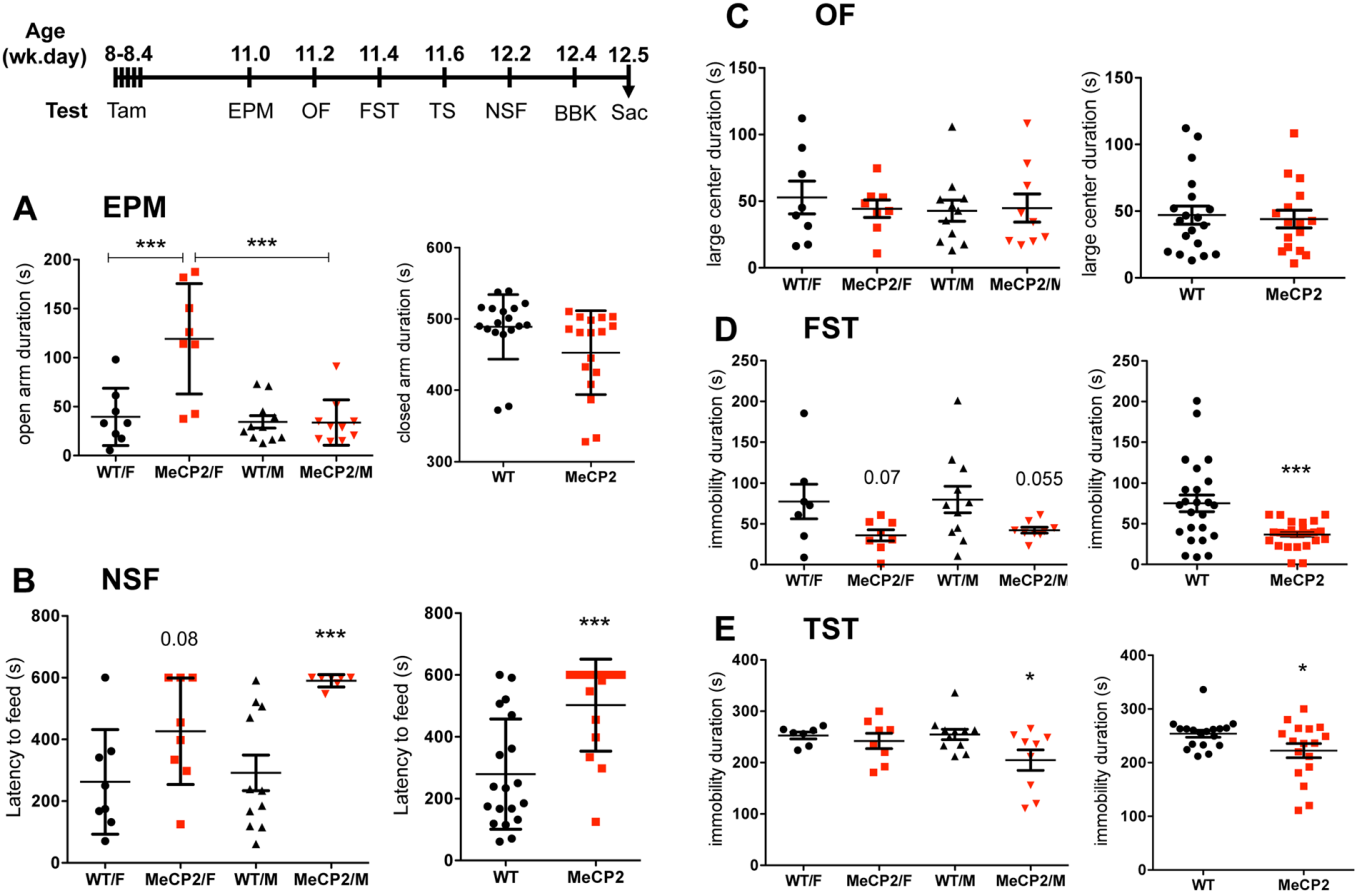
Sex-dependent changes in anxiety- and depression-like behavior in MeCP2 cKO mice. Mice were treated with tamoxifen (Tam), and subjected to behavioral tests according to the timeline shown and sacrificed one day after the last test (Sac). Male (M) and female (F) or pooled homozygous WT and MeCP2 cKO mice were compared (N = 8, WT/F and MeCP2/F; N = 11, WT/M; N = 9, MeCP2/M). (**A**) EPM test. Compared to WT, female MeCP2 mice spent more time in open arms (one-way ANOVA sex X genotype interaction, F3, 33 = 12.96, p < 0.001; post-hoc Tukey WT/F vs MeCP2/F ***p = 0.0002; MeCP2/F vs MeCP2/M p < 0.0001) with no difference detected in closed arm time, indicative of reduced anxiety in females. (**B**) NSF test. Male (one-way ANOVA sex X genotype interaction, F3, 30 = 6.702, p = 0.0014; post-hoc Tukey WT/M vs MeCP2/M ***p = 0.0032) and pooled MeCP2 cKO mice showed greater latency to approach food in the novel arena (WT, 279.4 ± 40.90 vs cko, 502.6 ± 38.44, t test, t = 3.892 df = 32, ***p = 0.0005), but no difference was seen in the home cage indicating increased anxiety. (**C**) FST test. Pooled MeCP2 cKO mice showed greater immobility time (WT, 75.15 ± 10.31 vs MeCP2 36.78 ± 3.204, t test, t = 3.615 df = 47, ***p < 0.0001, and males showed a strong trend (one-way ANOVA sex X genotype interaction F3, 31 = 2.791, p = 0.0569; post-hoc Tukey WT/M vs MeCP2/M, p = 0.055), indicating reduced depression-like behavior. (**D**) TST. Male (one-way ANOVA sex X genotype interaction F3, 31 = 4.080, p = 0.0149, post-hoc Tukey WT/M vs MeCP2/M, *p = 0.0164) and pooled MeCP2 cKO mice showed lower immobility time (WT, 254.0 ± 6.586 vs MeCP2, 222.4 ± 13.15, t test, t = 2.186 df = 33, *p = 0.0360), indicating reduced depression-like behavior in male MeCP2 cKO mice.

We have recently shown that specific de-repression of 5-HT1A autoreceptors upon knockout of the HTR1A repressor Freud-1/CC2D1A leads to anxiety- and depression-like behaviors^29^. We therefore tested these behaviors in the MeCP2 cKO mice according to the timeline shown (Fig. 4). Because the behavioral effects of MeCP2 mutations are often sex-dependent^50^, we also examined male and female mice separately. In the EPM test, female MeCP2 cKO mice showed increased time in the open arms, indicating reduced anxiety (Fig. 4A). Oppositely, in the NSF test, males showed increased latency to feed with the majority not approaching the food within 600 s, indicating strongly increased anxiety in this test that was great enough to result in a significant genotype-dependent increase in MeCP2 cKO mice (Fig. 4B). No differences between genotypes or sexes were seen in the OF anxiety test (Fig. 4C), nor in the BBK test, a control for locomotion activity (data not shown). These data suggest an opposite effect of the loss of MeCP2 in 5-HT neurons on anxiety in males and females. In the FST and the TS, the MeCP2 cKO mice showed reduced immobility times, driven mainly by the male mice (Fig. 4D,E). This suggests that loss of MeCP2 in 5-HT neurons results in reduced depression-like behaviors, especially in males. Thus in males, deletion of MeCP2 in 5-HT neurons leads to increased 5-HT1A autoreceptor levels that is associated with increased anxiety and reduced depressed-like behaviors, while in females an anti-anxiety phenotype is seen in the EPM.

## Discussion

### Deaf1-dependent recruitment of MeCP2 to the HTR1A promoter

Because both Deaf1 and DNA methylation have been implicated in HTR1A regulation, we examined their role in MeCP2 activity at the HTR1A promoter. Deaf1 binds the HTR1A promoter in an allele-specific manner, with the non-binding G(-1019) allele associated with major depression, anxiety and resistance to antidepressant and atypical antipsychotic treatment^31–33,51^. DNA methylation of the Deaf1 site is associated with negative symptoms of schizophrenia and correlates with antipsychotic resistance^47^. Using yeast one-hybrid and ChIP assays, we show that MeCP2 is recruited to this site indirectly via binding to Deaf1 and directly via CpG methylation of the site. We show that Deaf1 and MeCP2 co-precipitate, indicating that this recruitment likely involves Deaf1-MeCP2 protein interactions, although we cannot rule out involvement of other cofactors (e.g., co-repressor complexes^52–55^). Although mainly expressed in forebrain, MeCP2 was recruited to the Deaf1 sites of the mouse HTR1A promoter in the prefrontal cortex, hippocampus, and raphe-midbrain, implicating it in regulation of both pre- and post-synaptic 5-HT1A receptor expression. Interestingly, under basal (non-stressed) conditions, recruitment of MeCP2 to the HTR1A was entirely Deaf1-dependent and was absent in the Deaf1−/− mice. Thus, Deaf1 is required for recruitment of MeCP2 to regulate basal mouse HTR1A promoter activity in cells and *in vivo*.

### Deaf1 and MeCP2 coordinately regulate HTR1A expression

Transcriptional reporter assays in three cell models revealed a novel interaction between Deaf1 and MeCP2 activities on mouse and human HTR1A promoters. Mutation of the Deaf1 site led to a de-repression of HTR1A activity, preventing Deaf1, Hes1/5 or other repressor functions. Transfection of Deaf1 repressed HTR1A promoters, which was dependent on the C(-1019) human allele or Site 1 in the mouse HTR1A. In HEK-293 cells, Deaf1 displayed enhancer activity at the mouse HTR1A Site 2, identifying a new role for this site in Deaf1 action. Deaf1 has previously been shown to have repressor or enhancer activity depending on cell type and promoter, however the mechanisms determining Deaf1 activity remain unclear. In contrast to Deaf1, MeCP2 increased transcription of the mouse HTR1A in all cell lines, which required Deaf1 Site 1. However, at the human HTR1A promoter this effect was independent of Deaf1 (in Deaf1−/− MEFs) and the Deaf1 site (in the G(-1019) allele), suggesting that other MeCP2 mechanisms are involved. Importantly, co-transfection of MeCP2 and Deaf1 converted MeCP2 or Deaf1 enhancer effects into a strong repressor effect in all cell lines and this effect was dependent on the Deaf1 site in human and mouse (Site 1). This indicates that recruitment of MeCP2 by Deaf1 strengthens Deaf1-MeCP2 repression and prevents their enhancer functions, providing a novel mechanism for MeCP2 mediated repression via Deaf1.

### Role of MeCP2 in HTR1A expression *in vivo*

To address the function of MeCP2 in HTR1A regulation *in vivo* we generated MeCP2 cKO mice with targeted MeCP2 deletion in adult 5-HT neurons. We demonstrate that loss of MeCP2 increases 5-HT1A autoreceptor levels and function, consistent with a repressor function for MeCP2 in 5-HT neurons. Similarly, knockout of Deaf1 or conditional knockout of HTR1A repressor Freud-1/Cc2d1A in adult 5-HT neurons results in increased 5-HT1A autoreceptor levels and function in 5-HT neurons, while reducing raphe 5-HT levels^28,29,38^. In contrast, a recent study of MeCP2 -/y male mice did not observe changes in brainstem 5-HT1A receptor RNA, but may have missed changes in 5-HT neurons, which constitute a fraction of cells in brainstem^56^. In humans with Rett syndrome, inactivating mutations of MeCP2 result in an age-progressive reduction in CSF 5-HIAA, also seen in mice with lifelong knockout of MeCP2 in 5-HT neurons^7^. However, in the MeCP2-cKO mice there was no change in 5-HT or 5-HIAA in the raphe, hippocampus or prefrontal cortex (Fig. S3), suggesting a developmental effect of MeCP2 is required for this phenotype. Thus, knockout of these key HTR1A repressors each increased 5-HT1A autoreceptor expression and function. Although MeCP2 negatively regulates 5-HT1A autoreceptor expression in mice, it remains unknown whether 5-HT1A receptor dys-regulation also occurs in humans with Rett syndrome.

### Loss of MeCP2 in adult 5-HT neurons sex-dependently alters behavior

Behaviorally, MeCP2 cKO mice had a striking sex-dependent anxiety phenotype, which mirrors that in global MeCP2 knockout mice. Female MeCP2 cKO mice showed reduced anxiety in the EPM test, while male MeCP2 cKO mice showed increased anxiety in the NSF test. Similarly, female MeCP2+/− mice show reduced anxiety-like behavior in EPM and light-dark (LD) box tests^57^, suggesting an anxiogenic role for MeCP2 in 5-HT neurons in female mice. Oppositely, male MeCP2 −/y mice showed increased anxiety in the EPM, OF and LD tests^58^. The sex-dependent anxiety phenotype in our MeCP2 cKO mice matches that in global MeCP2 knockout mice, suggesting that loss of MeCP2 in 5-HT neurons may drive the anxiety phenotype in MeCP2 gene deletion models.

The reduced anxiety in females differs from the anxiety phenotype seen upon loss of HTR1A repressors Freud-1 and Deaf1. Loss of Freud-1 in adult 5-HT neurons increased anxiety- and depression-like behaviors in both sexes^29^. The increase in anxiety-like behavior in male MeCP2 cKO mice is similar to what is seen in Freud-1 cKO mice and implicates 5-HT1A autoreceptor up-regulation, which may partly account for anxiety in human male MeCP2 mutation carriers. In contrast, global Deaf1 −/− mice showed increased anxiety-like behavior that was sex- and test-dependent^28^. Deaf1 −/− females showed increased anxiety in the EPM and OF, but reduced anxiety in the light-dark test, while males showed increased anxiety in the light-dark test. In MeCP2 cKO mice, females showed reduced anxiety in the EPM, suggesting that MeCP2 has a Deaf1- and HTR1A-independent effect on female anxiety.

Our results suggest reduced depression-like behavior in males lacking MeCP2 in 5-HT neurons. Unlike other MeCP2 deletion mouse models^59,60^, we found no evidence for motor impairment in distance travelled in the BBK, OF or EPM tests, suggesting that the 5-HT system does not contribute to this phenotype. Due to the locomotor impairment, the FST is not feasible in MeCP2 -/y mice^61^, hence depression-like behavior has not been evaluated. Interestingly, patients with MeCP2 duplication display both anxiety and depression^5^. Conditional knockout of Freud-1 in mice lacking 5-HT1A autoreceptors elicited a similar anti-depressed phenotype as in MeCP2 cKO^29^. Since up-regulation of 5-HT1A receptors increases depression-like behavior, like Freud-1, MeCP2 may target other genes in 5-HT neurons to alter this phenotype.

The mechanisms underlying the sex-specific behavioral effect of MeCP2 are unclear. One major role of MeCP2 in gene regulation is sex-dependent parental imprinting by silencing of the methylated paternal chromosome^62,63^. In the brain, post-natal deletion of the chromatin remodeling protein ATR-X results in a loss of MeCP2-mediated inactivation and induction of imprinted genes in females^64^. A similar mechanism of female-specific gene regulation in adult 5-HT neurons could mediate the reduced anxiety in female MeCP2 cKO mice, but only if imprinted gene expression is reversible within 3 weeks of tamoxifen-induced MeCP2 knockout. Our results with HTR1A suggest that loss of MeCP2 can alter gene expression in this time frame.

## Conclusion

In summary, we have identified a novel MeCP2-Deaf1 interaction that enhances Deaf1 recruitment to the Deaf1 element of the HTR1A promoter, and switches on Deaf1-induced repressor activity. In the presence but not absence of Deaf1, both Deaf1 and MeCP2 bind to this site *in vivo*. Deaf1 and MeCP2 bind to the Deaf1 site in the mouse HTR1A promoter *in vivo*. Like Deaf1, knockout of MeCP2 enhanced 5-HT1A autoreceptor expression, suggesting that both play a role in HTR1A promoter regulation and contribute to anxiety or depression phenotypes. In males, MeCP2 in 5-HT neurons may mediate protection from anxiety but predisposition to depression, while in females, MeCP2 may drive anxiety.

## Methods

### Yeast one-hybrid

As described previously^30^, three repeats of either the C or G allele containing 26-bp Deaf1 element were fused to p8op-SN-LacZ, a β-galactosidase reporter plasmid (generously provided by Dr. Takahi Ito, Kanazawa University, Japan); the Gal4AD pACT2 plasmid was fused to Deaf1 (Clontech), and the pRS423 plasmid was fused to MeCP2 (Clontech). The PJ69-2A yeast strain (from Dr. Ito) was used to transform combinations of vectors including M.SssI (pLexA-MSssI), to analyse the effects of gene methylation^65^. β-galactosidase activity was measured using ortho-Nitrophenyl-β-D-galactopyranoside substrate.

### Cell lines

Human SKN-SH neuroblastoma, human HEK-293, and mouse Deaf1 +/+ and −/− mouse embryonic fibroblasts (MEFs) were grown in DMEM with 4.5 g/L glucose, without sodium pyruvate (Wisent) with 10% v/v heat-inactivated FBS (Wisent) at 37 °C and 5% CO_2_. Rat RN46A raphe cells were grown in Neurocell (Wisent) with 10% heat-inactivated FBS (Wisent) at 33 °C and 5% CO_2_^30^. To prepare MEFs, heterozygous Deaf1 females and males were bred and checked daily for plugs. Pregnant females were euthanized and MEFs isolated as described^66^ from embryos genotyped for Deaf1^38^. MEFs were immortalized using the pWP TS A58 lentiviral vector.

### Plasmid Constructs

As described previously^30^, 1128 C or 1128 G include 1128 bp upstream of the human 5-HT1A translational initiation site (TIS), with either the C or G (-1019) allele; the mouse 984 construct includes 984 bp upstream of the mouse TIS^38^. The WT1A, m1, and m1&m2 include 1438 bp upstream of the mouse 5HT1A TIS. In the m1 mutant 5′-CTCGTG is changed to 5′-TGCCGG and in the m1&m2 mutant 5′-GTCGAC of the second Deaf1 site is also replaced with 5′-GCGGCA. Transcription vectors included the pcDNA3-His-human Deaf1^30^ (denoted as Deaf1) or empty pcDNA3 vector and pFLAG-human MeCP2 (denoted as MeCP2) or the empty pcDNA3-FLAG (pFLAG) vector.

### Transfection and Luciferase Assays

MEFs grown to 90% confluence in a 5-cm dish (Corning) were transfected with 1.5 μg of Deaf1, MeCP2 and/or reporter plasmids or respective controls, 0.5 μg of β-Galactosidase and 10 μL of Fastfect (Feldan) for 5 μg total DNA. HEK-293 cells at 70% confluence in a 5 cm plate were transfected with 2.5 μg of each plasmid except for 0.5 μg of β-galactosidase using calcium-phosphate co-precipitation^67^. RN46A cells grown to 60% confluence in a Primaria (Falcon) 10-cm dish were transfected with 3 μg of each vector except for 0.5 μg of β-galactosidase using 19 μL of Lipofectamine 3000 and 5 μL of Plus reagent (ThermoFisher, Ottawa ON) for 9.5 μg of DNA (2:1 lipid:DNA ratio).

For luciferase assay, 48 hr after transfection cells were lysed with reporter lysis buffer (Promega) and luciferase luminescence read by a GloMax-96 microplate luminometer (Promega). β-galactosidase activity was measured using a LS50 spectrophotometer (Perkin-Elmer) and used to correct luciferase activity, which was then normalized to the appropriate pGL3B empty vector control and data expressed as a percentage of pGL3B activity. No significant difference in activity was found between pGL3B and empty vector controls.

### Co-immunoprecipitation

Lipofectamine 2000 (ThermoFisher) was used to transfect SKN-SH cells^42^ with pcDNA3-HIS-Deaf1 and pFLAG-MeCP2 or respective controls. All cells were grown to 80–90% confluence. Brain extracts were from 10 week-old mice and were minced. Samples were lysed with FLAG buffer (with protease inhibitors) and homogenized with 25 strokes of the large and small clearance pestle in a chilled 7 mL glass Dounce homogenizer. Lysates were centrifuged at 15000 rpm for 15 min at 4 °C and quantified. Lysates from transfected SKN-SH (500 μg/500 μL) were rotated for 1 hr with 100 μL of cobalt beads (Thermo Fisher; binds the HIS tag) or overnight (16 h) with 100 μL of anti-FLAG beads (Sigma) at 4 °C. Lysates of endogeneous proteins from cells and brains (1500 μg/1 mL) were incubated at 4 °C for 16 h with pre-immune IgG (1:50 v/v), anti-Deaf1 homemade rabbit antibody (1:50 v/v), or anti-MeCP2 rabbit antibody (Millipore CAT.# 07–013; 1:100 v/v), and incubated with 200 μL of protein A-Sepharose bead slurry (Sigma) per 1 mL of lysate for 2 h at 4 °C. Beads were then washed at 4 °C 5 times with 500 μL of FLAG lysis buffer and protease inhibitors, then resuspended in 1x Laemmli loading buffer (60 uL), boiled for 5 min, and centrifuged at 12000 g × 3 min to remove insoluble debris. Entire sample supernatants were loaded on Western blot, stained with either Anti-FLAG tag rabbit antibody (1:2000; Millipore); anti-Deaf1 rabbit polyclonal antibody against homemade recombinant full-length human Deaf1 (1:500 v/v); anti-MeCP2 chicken antibody (Millipore CAT.# ABE171; 1:1000 v/v); or anti-β-actin mouse antibody (Sigma), followed up by the appropriate HRP-conjugated (Sigma; 1:4000) secondary antibody, and HRP substrate (Millipore). Finally, these were detected with the Image Quant LAS4010 (GE) or Kodak film and presented in compliance with the digital image and integrity policies.

### Chromatin immunoprecipitation

Brain extracts from 10 week-old mice were minced and cells were grown to 80–90% confluence and processed as described previously^42^. However, samples were sonicated for 30 min at 4 °C using a 70 W, 40 KHz Ultra-sonic water bath (1.9 L) MH series, model 15-337-401 (ThermoFisher) and ChIP grade glass beads (0.1 g per 1 mL of lysate; Sigma). Beads were removed and supernatants (1500 μg/mL of ~500 bp DNA) were incubated with either pre-immune rabbit IgG (1:50), Deaf1 rabbit homemade antibody (1:50)^38^, MeCP2 rabbit antibody (1:100; Millipore CAT.# 07–013), or rabbit anti-Histone H1 (1:200, Abcam CAT.# AB31972) with constant rotation at 4 °C overnight. Samples were incubated for 2 h at 4 °C with 200 μL of Protein A-Sepharose beads (Sigma) in PBS, 250 μg/mL BSA and 100 μg/mL sonicated salmon sperm per 1 mL of sample. Samples were then washed, de-crosslinked and the DNA isolated as previously described^42^. PCR was performed using the mouse HTR1A promoter primers that encompass both Deaf1 binding sites (a 180-bp PCR product) and the mouse GAPDH promoter primers (a 179-bp PCR product)^38^. The PCR was performed using 200 μM dNTP, 1 μM of each primer, 2.5 U of Taq polymerase (NEB). Cycling conditions were: 95 °C for 1 min; 40 cycles of 95 °C for 30 sec, 59 °C (for HTR1A)/56 °C (for GAPDH) for 45 sec, 72 °C for 15 sec, and final extension at 72 °C, 5 min.

### Mice housing, breeding and genotyping

The University of Ottawa Animal Care Committee approved all animal protocols and all experiments were performed in accordance with University of Ottawa Animal Care Committee guidelines. Animals were housed with ad libitum food and water, on a 12-hour light/dark cycle (lights on at 6:00 AM), at 21 °C. TPH2Cre^ERT2^ (#016584) and MeCP2 (#007177) mice were obtained from Jackson Laboratories (Bar Harbor, ME) and interbred on a C57Bl/6 background. At 3 weeks of age an ear notch was taken for genotyping and extracted using the REDExtract-N-Amp Tissue PCR kit (Sigma). The PCR to genotype MeCP2 was done using red dye Taq PCR master mix (BioBasic) and 4 μM of the following primers: forward 5′-GCCACATGACAAGACAAAAACA; reverse 5′-GTTACACCG CTGAAATCTCTTG. The PCR conditions were: 94 °C for 4 min; 34 cycles of 94 °C for 30 sec, 57 °C for 30 sec, 72 °C for 30 sec; 72 °C for 10 min generating 238-bp (wild-type) or 313-bp (flx-MeCP2) products. TPH2Cre^ERT2^ PCR was performed using 1x EasyTaq buffer (Transgen Biotech), 200 μM dNTP, 0.05 U of EasyTaq polymerase (Transgen Biotech), 2 μM of each respective primers: 11679 5′-GCTGAGAAAGAAAATTACATCG and 12523 5′-TGGCTTGCAGGTACAGGAGG; and internal positive controls: OIMR8744 5′-CAAATGTTGCTTGTCTGGTG and OIMR8745 5′-GCTAGTCGAGTGCACAGTTT. Cycling conditions were: 94 °C for 1 min; 35 cycles of 94 °C for 15 sec, 57 °C for 20 sec, 72 °C for 10 sec; and 72 °C for 2 min with a 300-bp TPH2Cre^ERT2^ product and 200-bp internal positive control. Each mouse used in this study was genotyped at weaning and verified at sacrifice. For Cre activation, at 8 weeks of age mice were injected with tamoxifen (180 mg/kg/day, i.p; Sigma T56648) for four consecutive days. Using this protocol we obtain ~90% recombination specific to 5-HT cells^29^. Tamoxifen (30 mg/mL; Sigma CAT # T5648-5G) was prepared at 30 mg/ml in 1:9 ethanol:sunflower seed oil (Sigma).

### 5-HT1A level and function experiments

Co-immunofluorescence for 5-HT1A receptors and TPH, 5-HT1A receptor autoradiography and DPAT-induced hypothermia were performed and quantified as described previously^28,29^ using 10–11 week-old mice. For immunostaining; antibodies used included sheep anti-TPH, (Abcam, ab1541 1:100) and rabbit anti-5-HT1A receptor raised to the second intracellular loop of the 5-HT1A receptor (Cedarlane, Hornby, ON, Canada, 1:50)^38^. The secondary antibodies used were anti-sheep Cy3 (Jackson, 713-165-003, 1:200) and Alexa Fluor 488 anti-rabbit (ThermoFisher, A-21206, 1:1000). Images were acquired using Axiovision imaging software on a Zeiss Axio Observer D1 microscope (n = 3/group). Positive-stained cells were manually counted in every 4^th^ section (4/region; Bregma 4.36 to 4.72 mm) within a standardized template surrounding the structure of interest, using ImageJ 1.48 v software. 5-HT1A autoradiography of frozen 25-µm sections of dorsal raphe or hippocampus was done using ^125^I-MPPI (Perkin Elmer, Boston, MA) as previously described^28,68^ (n = 3). Signals were within the linear range of the film and quantified based on standard curve using ARC146-F ^14^C standards (American Radiochemicals Inc, St. Louis, MO). 8OH-DPAT-induced hypothermia was performed from 9–11 AM as described previously, with 3 baseline temperature measurements, administration of 8OH-DPAT (0.5 mg/kg, i.p., Sigma), followed by measurements at every 10 minutes^29^. The baseline values were averaged and the difference between the average baseline and recorded temperature was plotted across time.

### Behavioral studies

Behavioral tests were conducted in littermates starting 2 weeks after the last tamoxifen injection, at 11 weeks of age (see timeline, Fig. 4). Mice were housed under normal light conditions (lit from 6:00–18:00) and tests were performed beginning at 10:00, after at least 1 h of habituation to the testing room. Testing was performed as described previously^29^ and completed within 10 days in the following order: elevated plus maze (EPM); open field (OF); tail suspension (TS); forced swim test (FST); novelty suppressed feeding (NSF); and beam break test (BBK). All animals were of the same age at the start of testing and all tests were done in the above order and completed within 12 days. Each cohort included 10–32 mice/group. Throughout testing and behavioral analyses, the experimenter was blind to the mouse genotype.

### High Performance Liquid Chromatography (HPLC) Analysis

Levels of 5-HT and 5-HIAA were quantified in extracts of dissected tissues by HPLC^29^. For HPLC, MeCP2 KO and matched WT littermate mice (n = 4, 11 wks old) were sacrificed by cervical dislocation and decapitation.

### Statistical analyses

Graphs were made using Microsoft Excel and present means and standard error (SE) calculated. Statistical calculations (as specified in figure/table legends) were performed using Graphpad Prism 6.00 for Windows. For behavioral studies, no animals were excluded. For multiple comparisons, two-way ANOVA was used, with Bonferroni or Tukey post-test. Minimum significance was set at p < 0.05.

### Data availability

The datasets generated and/or analyzed during the current study are available from the corresponding author on reasonable request.

## Electronic supplementary material

Supplementary Information

## References

[1] Cassel S, Revel MO, Kelche C, Zwiller J (2004). Expression of the methyl-CpG-binding protein MeCP2 in rat brain. An ontogenetic study. Neurobiol Dis.

[2] Adachi M, Monteggia LM (2014). Decoding transcriptional repressor complexes in the adult central nervous system. Neuropharmacology.

[3] Chahrour M (2008). MeCP2, a key contributor to neurological disease, activates and represses transcription. Science.

[4] Amir RE (1999). Rett syndrome is caused by mutations in X-linked MECP2, encoding methyl-CpG-binding protein 2. Nat Genet.

[5] Ramocki MB (2009). Autism and other neuropsychiatric symptoms are prevalent in individuals with MeCP2 duplication syndrome. Annals of neurology.

[6] Temudo T (2009). Evaluation of CSF neurotransmitters and folate in 25 patients with Rett disorder and effects of treatment. Brain Dev.

[7] Samaco RC (2009). Loss of MeCP2 in aminergic neurons causes cell-autonomous defects in neurotransmitter synthesis and specific behavioral abnormalities. Proc Natl Acad Sci USA.

[8] Kessler RC, Bromet EJ (2013). The epidemiology of depression across cultures. Annual review of public health.

[9] Doris A, Ebmeier K, Shajahan P (1999). Depressive illness. Lancet.

[10] Sharp T, Cowen PJ (2011). 5-HT and depression: is the glass half-full?. Curr Opin Pharmacol.

[11] Stockmeier CA (2003). Involvement of serotonin in depression: evidence from postmortem and imaging studies of serotonin receptors and the serotonin transporter. J Psychiatr Res.

[12] Mann JJ (1999). Role of the serotonergic system in the pathogenesis of major depression and suicidal behavior. Neuropsychopharmacology.

[13] Jans LA, Riedel WJ, Markus CR, Blokland A (2007). Serotonergic vulnerability and depression: assumptions, experimental evidence and implications. Mol Psychiatry.

[14] Delgado PL (1999). Tryptophan-depletion challenge in depressed patients treated with desipramine or fluoxetine: implications for the role of serotonin in the mechanism of antidepressant action. Biol Psychiatry.

[15] Placidi GP (2001). Aggressivity, suicide attempts, and depression: relationship to cerebrospinal fluid monoamine metabolite levels. Biol Psychiatry.

[16] Lidberg L, Belfrage H, Bertilsson L, Evenden MM, Asberg M (2000). Suicide attempts and impulse control disorder are related to low cerebrospinal fluid 5-HIAA in mentally disordered violent offenders. Acta Psychiatr Scand.

[17] Albert PR, Lemonde S (2004). 5-HT1A Receptors, Gene Repression, and Depression: Guilt by Association. Neuroscientist.

[18] Stockmeier CA (1998). Increase in serotonin-1A autoreceptors in the midbrain of suicide victims with major depression-postmortem evidence for decreased serotonin activity. J Neurosci.

[19] Boldrini M, Underwood MD, Mann JJ, Arango V (2008). Serotonin-1A autoreceptor binding in the dorsal raphe nucleus of depressed suicides. J Psychiatr Res.

[20] Kaufman J (2015). Quantification of the Serotonin 1A Receptor Using PET: Identification of a Potential Biomarker of Major Depression in Males. Neuropsychopharmacology.

[21] Parsey RV (2010). Higher serotonin 1A binding in a second major depression cohort: modeling and reference region considerations. Biol Psychiatry.

[22] Savitz J, Lucki I, Drevets WC (2009). 5-HT(1A) receptor function in major depressive disorder. Prog Neurobiol.

[23] Bhagwagar Z, Rabiner EA, Sargent PA, Grasby PM, Cowen PJ (2004). Persistent reduction in brain serotonin1A receptor binding in recovered depressed men measured by positron emission tomography with [11C]WAY-100635. Mol Psychiatry.

[24] Lanzenberger RR (2007). Reduced serotonin-1A receptor binding in social anxiety disorder. Biol Psychiatry.

[25] Sullivan GM (2005). Brain serotonin1A receptor binding in major depression is related to psychic and somatic anxiety. Biol Psychiatry.

[26] Albert PR, Vahid-Ansari F, Luckhart C (2014). Serotonin-prefrontal cortical circuitry in anxiety and depression phenotypes: pivotal role of pre- and post-synaptic 5-HT1A receptor expression. Front Behav Neurosci.

[27] Garcia-Garcia AL, Newman-Tancredi A, Leonardo ED (2014). 5-HT(1A) receptors in mood and anxiety: recent insights into autoreceptor versus heteroreceptor function. Psychopharmacology (Berl).

[28] Luckhart C (2016). Sex-dependent adaptive changes in serotonin-1A autoreceptor function and anxiety in Deaf1-deficient mice. Mol Brain.

[29] Vahid-Ansari F (2017). Abrogated Freud-1/Cc2d1a Repression of 5-HT1A Autoreceptors Induces Fluoxetine-Resistant Anxiety/Depression-Like Behavior. J Neurosci.

[30] Lemonde S (2003). Impaired repression at a 5-hydroxytryptamine 1A receptor gene polymorphism associated with major depression and suicide. J Neurosci.

[31] Kishi T (2011). Serotonin 1A receptor gene, schizophrenia and bipolar disorder: An association study and meta-analysis. Psychiatry Res.

[32] Kishi T (2013). The serotonin 1A receptor gene confer susceptibility to mood disorders: results from an extended meta-analysis of patients with major depression and bipolar disorder. Eur Arch Psychiatry Clin Neurosci.

[33] Le Francois B, Czesak M, Steubl D, Albert PR (2008). Transcriptional regulation at a HTR1A polymorphism associated with mental illness. Neuropharmacology.

[34] Donaldson ZR (2016). The functional serotonin 1a receptor promoter polymorphism, rs6295, is associated with psychiatric illness and differences in transcription. Transl Psychiatry.

[35] Straube B (2014). The functional −1019C/G HTR1A polymorphism and mechanisms of fear. Transl Psychiatry.

[36] Czesak M, Lemonde S, Peterson EA, Rogaeva A, Albert PR (2006). Cell-specific repressor or enhancer activities of Deaf-1 at a serotonin 1A receptor gene polymorphism. J Neurosci.

[37] Hesselgrave N, Parsey RV (2013). Imaging the serotonin 1A receptor using [11C]WAY100635 in healthy controls and major depression. Philos Trans R Soc Lond B Biol Sci.

[38] Czesak M (2012). Increased serotonin-1A (5-HT1A) autoreceptor expression and reduced raphe serotonin levels in deformed epidermal autoregulatory factor-1 (Deaf-1) gene knock-out mice. J Biol Chem.

[39] Mekli K (2011). The HTR1A and HTR1B receptor genes influence stress-related information processing. Eur Neuropsychopharmacol.

[40] Samadi Rad B (2012). Serotonin 1A receptor genetic variations, suicide, and life events in the Iranian population. Psychiatry and Clinical Neurosciences.

[41] Vrshek-Schallhorn S (2015). Additive genetic risk from five serotonin system polymorphisms interacts with interpersonal stress to predict depression. Journal of abnormal psychology.

[42] Le Francois B (2015). Chronic mild stress and antidepressant treatment alter 5-HT1A receptor expression by modifying DNA methylation of a conserved Sp4 site. Neurobiol Dis.

[43] Turecki G, Meaney MJ (2016). Effects of the Social Environment and Stress on Glucocorticoid Receptor Gene Methylation: A Systematic Review. Biol Psychiatry.

[44] Klengel T (2013). Allele-specific FKBP5 DNA demethylation mediates gene-childhood trauma interactions. Nat Neurosci.

[45] McGowan PO (2009). Epigenetic regulation of the glucocorticoid receptor in human brain associates with childhood abuse. Nat Neurosci.

[46] Murgatroyd C (2009). Dynamic DNA methylation programs persistent adverse effects of early-life stress. Nat Neurosci.

[47] Tang H, Dalton CF, Srisawat U, Zhang ZJ, Reynolds GP (2014). Methylation at a transcription factor-binding site on the 5-HT1A receptor gene correlates with negative symptom treatment response in first episode schizophrenia. Int J Neuropsychopharmacol.

[48] Jensik PJ (2014). DEAF1 binds unmethylated and variably spaced CpG dinucleotide motifs. PLoS One.

[49] Pilot-Storck F (2010). Interactome mapping of the phosphatidylinositol 3-kinase-mammalian target of rapamycin pathway identifies deformed epidermal autoregulatory factor-1 as a new glycogen synthase kinase-3 interactor. Mol Cell Proteomics.

[50] Chahrour M, Zoghbi HY (2007). The story of Rett syndrome: from clinic to neurobiology. Neuron.

[51] Newman-Tancredi, A. & Albert, P. R. Gene polymorphism at serotonin 5-HT1A receptors: moving towards personalized medicine for psychosis and mood deficits. in *Schizophrenia Research: Recent Advances* (ed. Sumiyoshi, T.) 337–358 (Nova Publishers, New York, NY, 2012).

[52] Bird A (2008). The methyl-CpG-binding protein MeCP2 and neurological disease. Biochem Soc Trans.

[53] Ebert DH (2013). Activity-dependent phosphorylation of MeCP2 threonine 308 regulates interaction with NCoR. Nature.

[54] Lyst MJ (2013). Rett syndrome mutations abolish the interaction of MeCP2 with the NCoR/SMRT co-repressor. Nat Neurosci.

[55] Nott, A. *et al*. Histone deacetylase 3 associates with MeCP2 to regulate FOXO and social behavior. *Nat Neurosci* (2016). 10.1038/nn.4347 PMC5083138 27428650

[56] Vogelgesang S (2017). Analysis of the Serotonergic System in a Mouse Model of Rett Syndrome Reveals Unusual Upregulation of Serotonin Receptor 5b. Front Mol Neurosci.

[57] Samaco RC (2013). Female Mecp2(+/−) mice display robust behavioral deficits on two different genetic backgrounds providing a framework for pre-clinical studies. Human Molecular Genetics.

[58] McGill BE (2006). Enhanced anxiety and stress-induced corticosterone release are associated with increased Crh expression in a mouse model of Rett syndrome. Proc Natl Acad Sci USA.

[59] Chao HT (2010). Dysfunction in GABA signalling mediates autism-like stereotypies and Rett syndrome phenotypes. Nature.

[60] Meng, X. *et al*. Manipulations of MeCP2 in glutamatergic neurons highlight their contributions to Rett and other neurological disorders. *eLife* **5** (2016). 10.7554/eLife.14199 PMC4946906 27328325

[61] Stearns NA (2007). Behavioral and anatomical abnormalities in Mecp2 mutant mice: a model for Rett syndrome. Neuroscience.

[62] Li E, Beard C, Jaenisch R (1993). Role for DNA methylation in genomic imprinting. Nature.

[63] Ferguson-Smith AC, Sasaki H, Cattanach BM, Surani MA (1993). Parental-origin-specific epigenetic modification of the mouse H19 gene. Nature.

[64] Kernohan KD (2010). ATRX partners with cohesin and MeCP2 and contributes to developmental silencing of imprinted genes in the brain. Dev Cell.

[65] Feng SY, Ota K, Yamada Y, Sawabu N, Ito T (2004). A yeast one-hybrid system to detect methylation-dependent DNA-protein interactions. Biochem Biophys Res Commun.

[66] Xu, J. Preparation, culture, and immortalization of mouse embryonic fibroblasts. *Current protocols in molecular biolog*y **Chapter 2**8, Unit28.21 (2005). 10.1002/0471142727.mb2801s70 18265366

[67] Ou XM (2000). Novel dual repressor elements for neuronal cell-specific transcription of the rat 5-HT1A receptor gene. J Biol Chem.

[68] Donaldson ZR (2014). Developmental effects of serotonin 1A autoreceptors on anxiety and social behavior. Neuropsychopharmacology.

