# Supplementary material for: Loss of MeCP2 in adult 5-HT neurons induces 5-HT1A autoreceptors, with opposite sex-dependent anxiety and depression phenotypes
Source: Sci Rep. 2018 Apr 10;8:5788. doi: 10.1038/s41598-018-24167-8 (PMC5893553; doi:10.1038/s41598-018-24167-8)
Supplement: Supplementary file 1 — Supplementary Information [file 41598_2018_24167_MOESM1_ESM.pdf]

Loss of MeCP2 in adult 5-HT neurons induces 5-HT1A autoreceptors, with opposite sex-dependent anxiety and depression phenotypes

Tristan J. Philippe[#1,2], Faranak Vahid-Ansari[#1,2], Zoe R. Donaldson[3], Brice Le François[1], Amin Zahrai[1], Valérie Turcotte-Cardin[1,2], Mireille Daigle[1], Jonathan James[6], René Hen[4,5], Zul Merali[6], Paul R. Albert[1,2]*

SUPPLEMENTARY INFORMATION

Supplementary Table 1. Statistical analyses of activity of Deaf1, MeCP2 at the human HTR1A promoter.

Cell line	**HEK-293**		**Deaf1-/- MEF**		**RN46A**	
hHTR1A	**C**	**G**	**C**	**G**	**C**	**G**
**F(3, 11)**	7.94	1.27	26.15	8.218	49.69	77.54
**p**	**0.009**	0.3081	**<0.0001**	**0.0042**	**<0.0001**	**<0.0001**

Cell line	**HEK-293**		**Deaf1-/- MEF**		**RN46A**	
hHTR1A	**C**	**G**	**C**	**G**	**C**	**G**
**MeCP2**	0.9994	N/A	**0.0129**	**0.0081**	**0.0109**	**0.0002**
**Deaf1**	**0.0008**	N/A	**0.0059**	0.2442	**<0.0001**	**0.0246**
**Deaf1-MeCP2**	**0.0063**	N/A	**0.0001**	0.0914	**<0.0001**	**<0.0001**

	**HEK-293**	**Deaf1-/- MEF**	**RN46A**
**MeCP2**	0.0828	**<0.0001**	**<0.0001**
**Deaf1**	0.8837	0.0769	0.5265

Statistical analyses used repeated measures one-way ANOVA **(top)** to analyse the effects of transcription factors on expression vector activity. Post-hoc analyses to compare empty vector controls and transcription factors using Dunnett's multiple comparison **(middle)** were performed when the main effect was $p<0.05$. Post-hoc to compare the effects of MeCP2 and Deaf1 to MeCP2 or Deaf1 alone on the 1128C vector were performed using Tukey's post-hoc test **(bottom).** Bold indicates statistical significance.

Supplementary Table 2. Statistical analyses of the activity of Deaf1 and MeCP2 at the mouse HTR1A promoter.

Cell line	HEK-293			Deaf1 -/- MEF			RN46A		
Mouse HTR1A	WT	m1	m1m2	WT	m1	m1m2	WT	m1	m1m2
F (3, 11)	27.61	31.04	15.3	47.41	5.38	5.649	108.8	7.696	0.3973
p	**<0.0001**	**<0.0001**	**0.0002**	**<0.0001**	**0.0125**	**0.0184**	**<0.0001**	**0.0058**	0.5813

	HEK-293	Deaf1 -/- MEF	RN46A
MeCP2	**0.0001**	**<0.0001**	**<0.0001**
Deaf1	**0.0001**	**<0.0001**	**0.0351**

Cell line	HEK-293			Deaf1 -/- MEF			RN46A		
Mouse HTR1A	WT	m1	m1m2	WT	m1	m1m2	WT	m1	m1m2
MeCP2	**0.0014**	0.8957	**0.0002**	**0.0004**	>0.9999	0.1333	**0.0002**	**0.0046**	N/A
Deaf1	**0.0006**	**<0.0001**	>0.9999	**0.0002**	0.0501	0.0632	**<0.0001**	0.1274	N/A
Deaf1-MeCP2	**0.0254**	0.5586	**0.0008**	**<0.0001**	0.0701	0.1097	**<0.0001**	0.3740	N/A

Statistical analyses used repeated measures one-way ANOVA **(top)** to analyse the effects of transcription factors on mouse HTR1A wild-type (WT), mutant m1 (m1) or both (m1m2) luciferase expression vector activity. Post-hoc analyses to compare empty vector controls and transcription factors (Deaf1, MeCP2 or both Deaf1-MeCP2 using Dunnett's multiple comparison **(middle)** were performed when the main effect was p<0.05. Post-hoc to compare the effects of

MeCP2 and Deaf1 to MeCP2 or Deaf1 alone on the WT vector were performed using Tukey's post-hoc test **(bottom).** Bold indicates statistical significance.

Supplementary Figures

Figure S1. Endogenous MeCP2 co-precipitates with Deaf1, only when Deaf1 is present. Co-IPs were performed using a rabbit pre-immune IgG as background control (Ig), Deaf1 (D1), and MeCP2 (M2) antibodies respectively and 1μg/μL of **(A) SKN-SH cell extract, (B) Deaf1 +/+ and -/- MEF cell extract**, **(C)** homogenized **Hippocampal** (hippo) tissue extract from several 10 week old Deaf1 WT or KO mice. Pull downs and inputs (in) were loaded on Western blot. Similar amounts of proteins were co-IPed (β-actin). The MeCP2 western was stained with 1:1000 chicken IgY against MeCP2 (Millipore CAT.# ABE171) and then with 1:4000 of a HRP-conjugated anti-chicken secondary antibody. MW (kDa) is shown.

Figure S2. Deaf1 and MeCP2 bind to the mouse 5-HT1A gene promoter region in a Deaf1 dependent fashion in brain tissues. The (A) Raphe, (B) hippocampus (Hippo), or (C) PFC areas were dissected from seven 10 week old mice, either wild-type (Deaf1 +/+) or knockout (Deaf1 -/-) and pulled down with either pre-immune rabbit IgG (Ig), Deaf1 (D1) antibody or MeCP2 (Me) antibody (Millipore CAT.# 07-013). As shown in Fig. 3, 300 bp, 200 bp and 100 bp bands of the molecular size ladder (MW), input (in) and negative (buffer) control for PCR amplification (–) are shown.

Figure S3. 5-HT and 5-HIAA content in MeCP2 cKO vs. WT mice. Tissue 5-HT and 5-HIAA content in prefrontal cortex (PFC), hippocampus (Hippo) and dorsal raphe was quantified by HPLC. Data represent mean ± SEM, n=3/group.

Figure S1

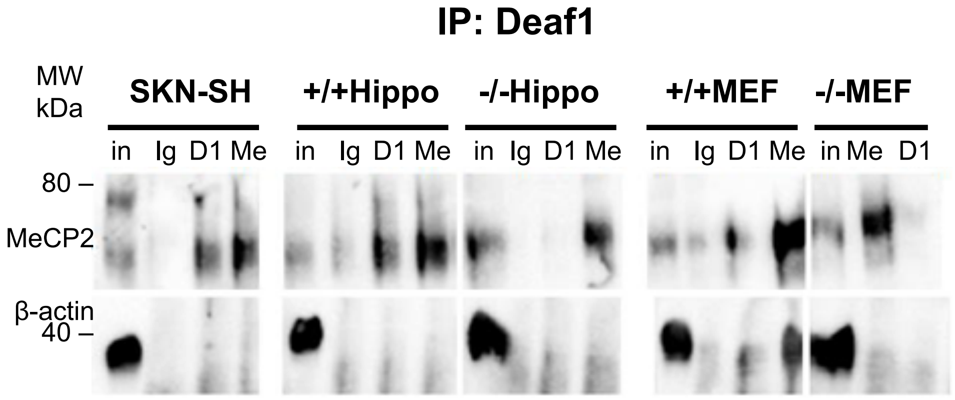

IP: Deaf1
MW
kDa
SKN-SH
+/+Hippo
-/-Hippo
+/+MEF
-/-MEF
in Ig D1 Me
in Ig D1 Me
in Ig D1 Me
in Ig D1 Me
in Me D1
80 –
MeCP2
β-actin
40 –

Figure S2

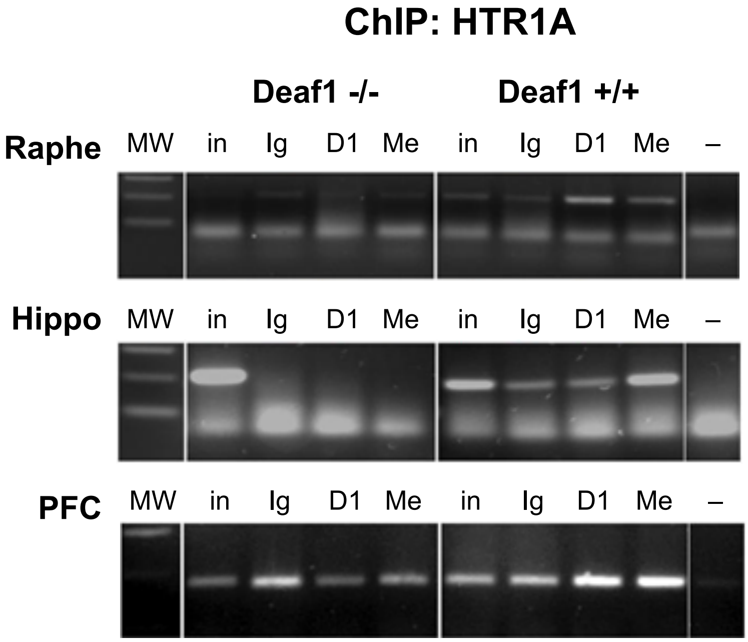

ChIP: HTR1A
Deaf1 -/-
Deaf1 +/+
Raphe
MW in Ig D1 Me in Ig D1 Me –
Hippo
MW in Ig D1 Me in Ig D1 Me –
PFC
MW in Ig D1 Me in Ig D1 Me –

Figure S3

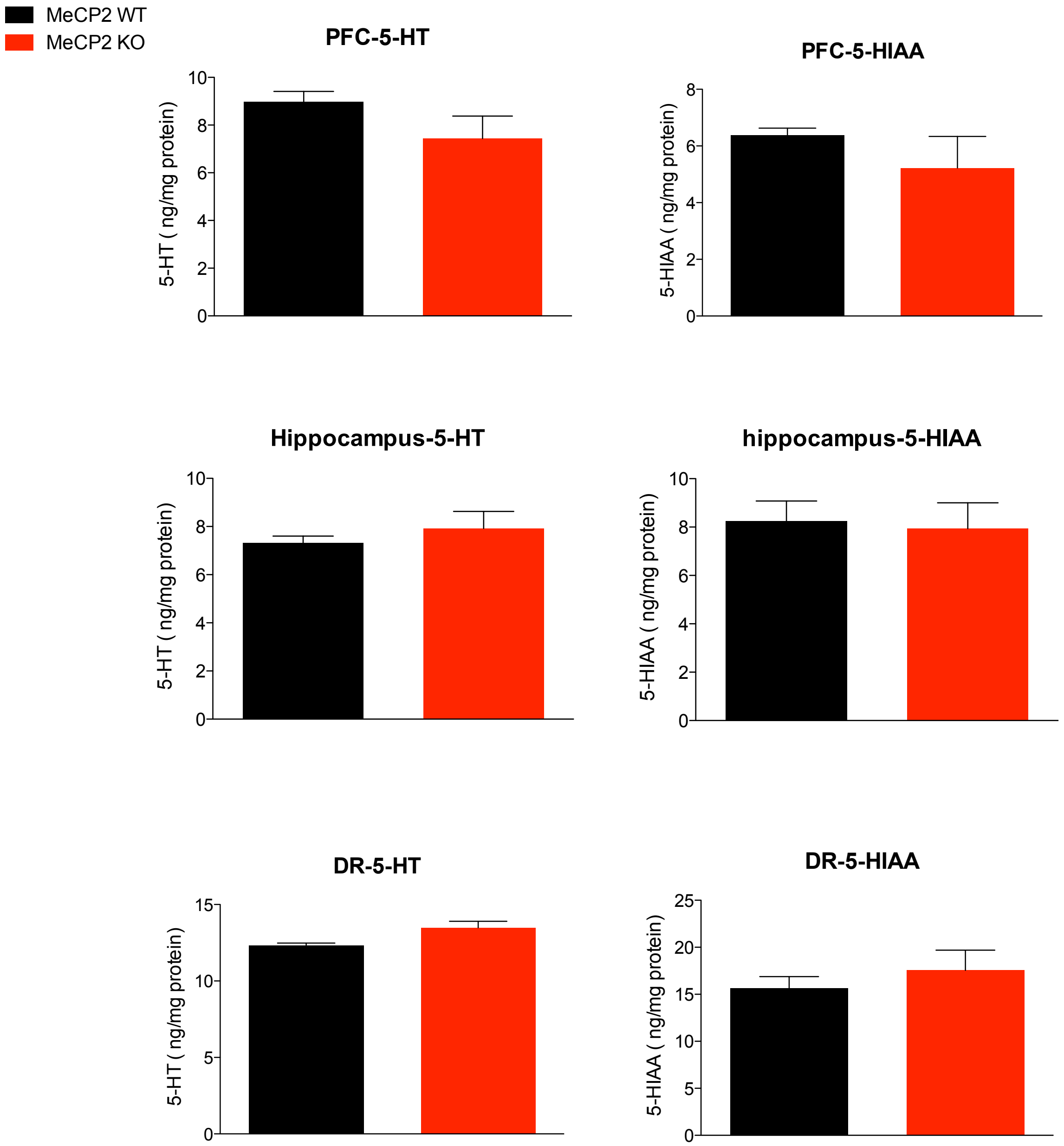
MeCP2 WT
MeCP2 KO
PFC-5-HT
5-HT (ng/mg protein)
PFC-5-HIAA
5-HIAA (ng/mg protein)
Hippocampus-5-HT
5-HT (ng/mg protein)
hippocampus-5-HIAA
5-HIAA (ng/mg protein)
DR-5-HT
5-HT (ng/mg protein)
DR-5-HIAA
5-HIAA (ng/mg protein)
